# Anxiety and procrastination in distance learning

**DOI:** 10.1192/j.eurpsy.2021.811

**Published:** 2021-08-13

**Authors:** D. Boyarinov, Y. Novikova, L. Gubaidulina, F. Sultanova, A. Kachina, V. Barabanshchikova

**Affiliations:** Faculty Of Psychology, Lomonosov Moscow State University, Moscow, Russian Federation

**Keywords:** Anxiety, Procrastination, Distance learning, students

## Abstract

**Introduction:**

In the context of distance learning students have an increase in the level of stress, anxiety (Husky, Kovess-Masfety, Swendsen, 2020). There is also a problem with time management and, as a result, procrastination. The reported study was funded by RFBR according to the research project №20-04-60174.

**Objectives:**

To study the differences in the level of anxiety and procrastination depending on the type of learning.

**Methods:**

A total of 290 students took part in the study. In the first study (before distance), 168 people took part, the average age was 19.8. In the second study (during distance) – 120 students, the average age was 19.2. The questionnaires: General Procrastination Scale, C.Lay; State-Trait Anxiety Inventory, Ch.Spielberger.

**Results:**

In the course of descriptive statistics, it was revealed that the level of procrastination and state anxiety have a middle score. However, the level of trait anxiety in conditions of distance learning is high, especially among 1st-year students. In a comparative analysis of the two studies, it turned out that the level of state anxiety is significantly higher (t=1,975;p=0,049) in conditions of distance learning. The correlation analysis revealed the relationship between procrastination and trait anxiety (r=0,414;p=0,0001).

**Conclusions:**

These results can be used to create programs to optimize the stress manifestation in students, especially when taking online exams. The high anxiety of 1st-year students may be associated with their accumulated stress factors, such as uncertainty about the future and etc. It should be noted that the level of procrastination does not differ, which may indicate procrastination as a personality trait.

